# Vienna-PTM web server: a toolkit for MD simulations of protein post-translational modifications

**DOI:** 10.1093/nar/gkt416

**Published:** 2013-05-22

**Authors:** Christian Margreitter, Drazen Petrov, Bojan Zagrovic

**Affiliations:** ^1^Institute for Molecular Modeling and Simulation, University of Natural Resources and Life Sciences, Muthgasse 18, 1190 Vienna, Austria and ^2^Max F. Perutz Laboratories, University of Vienna, Campus Vienna Biocenter 5, 1030 Vienna, Austria

## Abstract

Post-translational modifications (PTMs) play a key role in numerous cellular processes by directly affecting structure, dynamics and interaction networks of target proteins. Despite their importance, our understanding of protein PTMs at the atomistic level is still largely incomplete. Molecular dynamics (MD) simulations, which provide high-resolution insight into biomolecular function and underlying mechanisms, are in principle ideally suited to tackle this problem. However, because of the challenges associated with the development of novel MD parameters and a general lack of suitable computational tools for incorporating PTMs in target protein structures, MD simulations of post-translationally modified proteins have historically lagged significantly behind the studies of unmodified proteins. Here, we present Vienna-PTM web server (http://vienna-ptm.univie.ac.at), a platform for automated introduction of PTMs of choice to protein 3D structures (PDB files) in a user-friendly visual environment. With 256 different enzymatic and non-enzymatic PTMs available, the server performs geometrically realistic introduction of modifications at sites of interests, as well as subsequent energy minimization. Finally, the server makes available force field parameters and input files needed to run MD simulations of modified proteins within the framework of the widely used GROMOS 54A7 and 45A3 force fields and GROMACS simulation package.

## INTRODUCTION

Post-translational modifications (PTMs) of proteins, such as phosphorylation, acetylation, methylation, carboxylation or hydroxylation, play a key role in a variety of different cellular processes (1,2). For example, PTMs have been shown to be important in regulating enzyme activity (1,2), ensuring proper localization of biomolecules (3,4), modifying protein stability (5,6) or directing chromatin remodeling (6,7). What is more, non-enzymatic PTMs, such as carbonylation or oxidation, frequently arise as a consequence of oxidative stress and are considered to be a ubiquitous mode of non-specific protein damage (8,9) involved in age-related disorders including neurodegenerative diseases, cancer and diabetes. Importantly, amino acids often undergo a significant change in their physico-chemical properties on modification, resulting sometimes in a dramatic alteration of the structure of the affected protein, its dynamics and the way it interacts with the environment (1,10–13). Of the 20 canonical amino acids, 17 can be modified, thus creating a vast source of proteome diversification. The paramount importance of such modifications is underscored by the fact that ∼5% of the human genome encodes enzymes related to PTMs (1). However, despite their extreme biological relevance, our atomistic-level understanding of PTMs and their effect on protein structure, dynamics and interaction networks is still rudimentary.

Molecular dynamics (MD) computer simulations using semi-empirical atomistic force fields are a powerful way to study biomolecules at a single-molecule level with atomistic spatial resolution and femtosecond temporal resolution (14,15). In particular, MD simulations allow one to study properties and processes that are not directly accessible through experiment and frequently play an important role in interpreting time- and ensemble-averaged experimental results (15,16). What is more, the power of MD simulations in particular and computational modeling approaches in general is expected to only increase in the future because of growing computational capabilities and ever-improving models. Despite this inherent potential, simulation studies of PTMs have typically lagged behind both wet-laboratory research and simulation studies of unmodified proteins, focusing even in best cases only on a few modification types for a small subset of proteins (9,10,12,17–22) (G. A. Khoury, J. P. Thompson and C. A. Floudas, unpublished results). The reasons for this are 2-fold. First, there are currently no computational tools allowing one to quickly and accurately modify protein structures with PTMs of choice, a necessary prerequisite for any MD simulations of PTMs. Second, there are no self-consistent, comprehensive force field parameters for treating the large majority of protein PTMs in MD simulations. Although there exist several automated or semi-automated tools for generating MD parameters for novel groups, such as ParamChem or SwissParam for CHARMM (23–26), q4md-forcefieldtools for AMBER/GLYCAM (27,28) or ATB for GROMOS (29) force fields, none of them focuses exclusively on PTMs or provides human-curated and validated PTM parameters.

**Figure 1.**
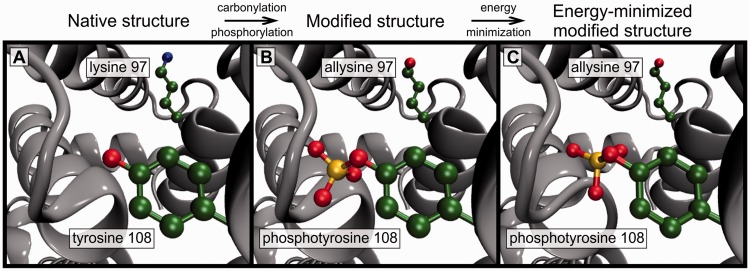
Vienna-PTM web server workflow. The server introduces one or more PTMs of choice to a user-supplied PDB structure followed by optional energy minimization. In the example, carbonylation and phosphorylation modifications are added to two select residues in human serum albumine [PDB code: 1N5U (33)].

This article focuses on the first of the aforementioned challenges by presenting Vienna-PTM web server (http://vienna-ptm.univie.ac.at), a web-based platform for introducing PTMs of choice in Protein Data Bank (PDB) structures (30) and GROMACS (31,32) structure files quickly and in a realistic fashion. Practically, adding PTMs to a structure of choice entails altering the chemical composition of select residues, including deletion of unnecessary atoms, geometrically and energetically realistic addition of new atoms, renumbering of atomic indices and residue renaming. In particular, addition of new atoms to a structure can take considerable effort, as the appropriate atomic coordinates have to be determined for each individual modified amino acid, and any inconsistency with force field definitions may lead to severe problems. To assist with this, Vienna-PTM web server provides an automated protein structure modification procedure, including 256 chemically distinct PTM reactions whereby users are able to give their instructions through an intuitive graphical interface, limiting errors to a minimum (the workflow of the server is illustrated in Figure 1). The required time from the initial PDB structure to the energy-minimized altered structure of choice can thus be reduced to several seconds. Finally, as a repository of newly developed PTM parameters (D. Petrov, C. Margreitter, M. Grandits, C. Oostenbrink and B. Zagrovic, under review) for two widely used and extensively tested MD force fields [GROMOS 45A3 (34) and 54A7 (35,36)], the server also directly addresses the second challenge aforementioned. In particular, in addition to modified PDB files, the output of the server includes all relevant structure and topology files needed to run MD simulations of modified proteins using GROMACS biomolecular simulation package and one of the aforementioned two force fields.

## MATERIALS AND METHODS

### Vienna-PTM web server: input data and overall workflow

**Figure 2.**
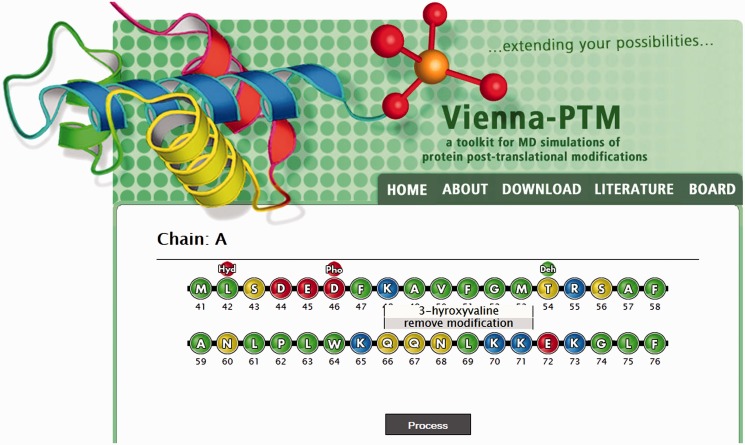
Data input. Modifications of choice are specified via a user-friendly graphical interface (depicted) or an optional text-based interface.

The input data that are passed to the server consists of (i) a protein X-ray or nuclear magnetic resonance structure in the form of a code-specified or manually uploaded PDB file together with processing options, such as MD force field and energy-minimization specification, and (ii) a choice of residues to be modified together with desired modifications. On upload of a protein structure in Step 1, the server redirects users to a page with the sequence from the uploaded PDB file interactively displayed either as ‘pearls on a string’ (Figure 2, graphical interface, JavaScript required) or a list (text-based interface) with available modifications for each residue given in drop-down menus. Depending on the interface type, selected modifications are either collected in a hidden text field or forwarded separately. In the graphical interface, a modified residue is visually labeled with a modification mark (Figure 2). When a job is submitted, the server adds, deletes, renames and renumbers atoms to apply the selected PTM(s), followed by an optional energy-minimization/geometry optimization. To maximize input coverage, all statements in the input PDB file except ATOM lines are ignored in the main modification step. This also means that already modified proteins may be uploaded again. Non-canonical residues in the input PDB file are represented as an exclamation mark and cannot be modified. Finally, if one uses a nuclear magnetic resonance structure with multiple model structures as input, the server modifies just the first model and includes it in the modified PDB file. In addition, a notification is issued on the results page informing the user of this fact. Detailed instructions can be obtained on server webpage (http://vienna-ptm.univie.ac.at/about.php), whereas support requests and reports of problems can be communicated in a user board (http://vienna-ptm.univie.ac.at/wbb).

Writing configuration files, calling back-end modules and checking status of current jobs (every few seconds) are carried out by back-end processing scripts. The back-end module provides parameters for each particular modification and force field combination. New atoms are added using relative pre-minimized coordinates for the modified side chain in a coordinate system whose axes are defined taking the last remaining bond, the reference point and the last dihedral orientation into account to avoid unfavorable side-chain conformations. The modification step itself takes ∼9 s on average, with minimization up to 3 min for largest systems. On the final result page, job-related information, such as the log file, is displayed to the user together with download links.

### Output

The final output of the server includes (i) a three-letter-code sequence of the modified protein, (ii) a PTM-containing PDB file [visualized on the webpage using Jmol (37)], (iii) GROMACS MD simulation input files and force field parameters for simulating the modified protein, including the GROMACS structure file (.gro) and topology file (.top) and (iv) modification and energy minimization log files. In the output PDB file, the modified residues are treated the same as canonical ones, meaning that they are added in the ATOM instead of the HETATM section of the resulting PDB file. Moreover, the original HETATM entries are also included in the output and renumbered properly, together with chain information and MODEL/ENDMDL statements. Depending on user’s specifications, the original header information, including REMARK and COMPND fields, is also included in the output file. Note that if energy minimization is not chosen during initialization, a modified PDB file is produced without the associated GROMACS files. Finally, one should emphasize that the server only provides files needed to prepare and run MD simulations, but not computational resources to do so.

### Features of the web server

#### Handling of input files

Input files can be either uploaded from a local hard drive or specified by a PDB ID. In the latter case, the PDB file is automatically downloaded from http://www.pdb.org. On user’s request on the initialization page, header information may be copied (not parsed) to the output PDB file. Depending on a modification, some information given in the original header may not be consistent with the modified structure; therefore, this option should be used with caution.

#### Available modifications

The server currently covers 256 distinct PTM reactions, including phosphorylation, methylation, acetylation, hydroxylation, carboxylation, carbonylation, nitration, deamidation and many others or 110 non-redundant post-translationally modified amino acids and protein termini. The difference between these two figures arises from the fact that a number of PTM reactions result in the same final modification. The complete list of all available modifications and the associated chemical structures are given in Supplementary Materials, whereas the details of the parameter development and the results of the validation procedure are further discussed in reference (D. Petrov, C. Margreitter, M. Grandits, C. Oostenbrink and B. Zagrovic, under review). In principle, the number of modifications that can be applied simultaneously is limited only by the size of the protein. However, if a large number of simultaneous modifications are requested in combination with energy minimization, the time limit for a particular job may be exceeded in rare cases. If this occurs, the final output contains an unminimized structure. Finally, in all output files, the newly introduced modified residues are represented using a three-letter residue code to match PDB file format definitions (version considered: 3.20).

#### Energy minimization

All modified residues have been pre–energy-minimized using the GROMOS 45A3 (34) or 54A7 (35,36) force fields before being incorporated into the target protein. To optimize the geometry and energy of the entire modified protein, energy minimization may be requested during the initialization step. In such cases, an initial test is performed to check whether the uploaded file is suitable as input for minimization. A negative outcome leads automatically to deactivation of minimization. The reasons for failure can be inclusion of non-standard residues or unique ligands, missing atoms or residues, non-standard formatting of PDB file and others. It is the responsibility of the user to provide a suitable PDB file for energy minimization. Note, however, that the initial coordinates of newly added atoms have been pre-minimized, thus ensuring meaningful initial coordinates even if minimization of the whole molecule is disabled or fails. Energy minimization uses GROMACS routines to perform steepest gradient minimization: 1500 minimization steps are performed *in vacuo* with a maximum force convergence threshold of 1.0 kJ/mol/nm. A cut-off range of 1.4 nm is used for both the van-der-Waals and Coulomb interactions. The .mdp files used for minimization are available for download in the ‘DOWNLOAD’ section of the server (http://viennaptm.univie.ac.at/download.php).

#### Security.

Job files cannot be downloaded or deleted (both uploaded and rendered) without the correct passphrase, which is generated automatically. This key is provided implicitly in links and, in case this is specified, sent to the user by email. Note that no email will be sent in case the job gets aborted. Once the job has been deleted, there is no way to recover data.

### Technical details

Vienna-PTM runs on a dedicated web server with sufficient storage capacity for ∼18 000 jobs. At the moment, eight jobs can run in parallel. The job limit is due to the fact that both the server and the modification programs are executed on the same physical machine. The server software is Apache2. The front end is written using MySQL, PHP5, JavaScript, CSS and Jmol plugin (37), whereas the back end is written in C++ (OO). GROMACS version 4.5.5 is used for energy minimization.

## CONCLUSIONS

Vienna-PTM is a freely available tool, which allows rapid and reliable addition of a wide variety of PTMs to protein side chains and termini. The workflow of the server results in an output PDB file, which can be downloaded and used for simulation studies or visualization purposes. By also including molecular dynamics parameters for modified amino acids and relevant input files, Vienna-PTM web server also provides a comprehensive platform to support all key steps in setting up MD simulations of post-translationally modified proteins. The parameters are currently available for GROMOS force fields 45a3 (34) and 54a7 (35,36) and are provided in GROMACS file formats both for versions <4.5.x and ≥4.5.x (31,32). Addition of new modification types and even completely new force fields to the server is logistically straightforward because of its flexible structure. Although MD parameters for several different PTMs have been developed and used before (9,10,12,17–22) (G. A. Khoury, J. P. Thompson and C. A. Floudas, unpublished results), Vienna-PTM is to the best of our knowledge the first publicly available repository containing human-curated and validated parameters for an almost complete set of biologically relevant modifications.

The Vienna-PTM server was launched in June 2012 for testing purposes and is expected to have high visibility in MD and PTM research communities. The focus in web design was on compatibility, preferably almost independent of the user’s operating system and browser settings. In conjunction with extensive beta-testing (altogether, ∼3000 test jobs have been performed by the authors and another 1000 by external beta-testers), this ensures maximal stability and user-friendliness. From direct MD simulations to biomolecular structure refinement to computational free-energy estimation and drug design, Vienna-PTM web server greatly expands the range of MD methodology to a large class of biomolecular systems of paramount importance. It is our hope that this advance will further catalyze the usage of analytical, quantitative methods of structural biophysics and chemistry, as embodied in the MD method, in addressing questions concerning realistic, PTM-dominated cell biology.

## SUPPLEMENTARY DATA

Supplementary Data are available at NAR Online: Supplementary Table 1.
